# 4-Nitro-6-[(8-quinolyl­amino)­methyl­idene]cyclo­hexa-2,4-dien-1-one

**DOI:** 10.1107/S1600536810025250

**Published:** 2010-07-03

**Authors:** Takashi Shibahara, Kentaro Takano, Masayuki Takahashi, Mikio Yamasaki

**Affiliations:** a Department of Chemistry, Okayama University of Science, Ridai-cho, Kita-ku, Okayama 700-0005, Japan; b Rigaku Corporation, 3-9-12, Matsubara-cho, Akishima-shi, Tokyo 196-8666, Japan

## Abstract

The mol­ecule of the title compound, C_16_H_11_N_3_O_3_, exists in the keto form and the C=O and N—H bonds are mutually *cis* in the crystal structure. There are two crystallographically independent mol­ecules per asymmetric unit with broadly similar structural data. The only noticeable difference between the two is the dihedral angles between the benzene and the quinoline rings: 1.04 (4) and 3.07 (4)°. In the structure, intra­molecular N—H⋯O(carbon­yl) and N—H⋯N(pyridine) hydrogen bonds exist but there is no evidence of formal inter­molecular hydrogen-bonding associations.

## Related literature

For a related structure, see: Shibahara *et al.* (2010 ▶).
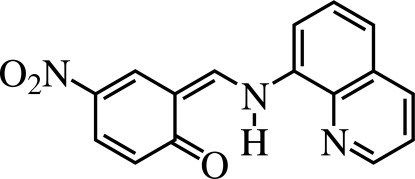


## Experimental

### 

#### Crystal data

C_16_H_11_N_3_O_3_
*M* *_r_* = 293.28
Triclinic, 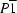

*a* = 7.1583 (6) Å
*b* = 8.2978 (7) Å
*c* = 22.040 (2) Å
α = 87.432 (9)°
β = 86.258 (12)°
γ = 83.518 (11)°
*V* = 1297.1 (2) Å^3^
*Z* = 4
Mo *K*α radiation
μ = 0.11 mm^−1^
*T* = 93 K
0.25 × 0.23 × 0.18 mm

#### Data collection

Rigaku Mercury70 CCD diffractometer
Absorption correction: multi-scan (*REQAB*; Jacobson, 1998 ▶) *T*_min_ = 0.881, *T*_max_ = 0.981
16456 measured reflections
5854 independent reflections
4472 reflections with *F* ^2^ > 2σ(*F* ^2^)
*R*_int_ = 0.040

#### Refinement

*R*[*F* ^2^ > 2σ(*F* ^2^)] = 0.043
*wR*(*F* ^2^) = 0.130
*S* = 1.06
5854 reflections
397 parameters
H-atom parameters constrained
Δρ_max_ = 0.32 e Å^−3^
Δρ_min_ = −0.33 e Å^−3^

### 

Data collection: *CrystalClear-SM Expert* (Rigaku, 1999 ▶); cell refinement: *CrystalClear-SM Expert*; data reduction: *CrystalClear-SM Expert*; program(s) used to solve structure: *SIR2004* (Burla *et al.*, 2005 ▶); program(s) used to refine structure: *SHELXL97* (Sheldrick, 2008 ▶); molecular graphics: *CrystalStructure* (Rigaku, 2007 ▶); software used to prepare material for publication: *CrystalStructure*.

## Supplementary Material

Crystal structure: contains datablocks global, I. DOI: 10.1107/S1600536810025250/jh2173sup1.cif

Structure factors: contains datablocks I. DOI: 10.1107/S1600536810025250/jh2173Isup2.hkl

Additional supplementary materials:  crystallographic information; 3D view; checkCIF report

## Figures and Tables

**Table 1:** Hydrogen-bond geometry (Å, °)

*D*—H⋯*A*	*D*—H	H⋯*A*	*D*⋯*A*	*D*—H⋯*A*
N2—H2N⋯O1	0.88	2.01	2.6849 (15)	132
N2—H2N⋯N3	0.88	2.31	2.6942 (16)	107
N5—H5N⋯O6	0.88	1.88	2.5915 (14)	137
N5—H5N⋯N6	0.88	2.25	2.6645 (15)	109

**Fig. 1.**
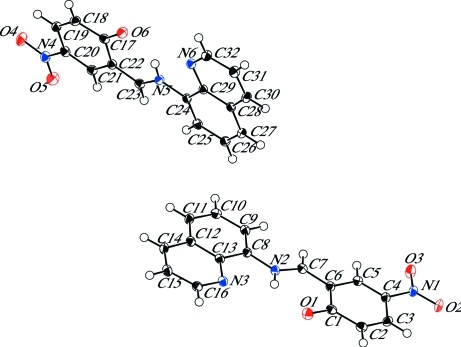
Molecular configuration and atom-numbering scheme for (I) with displacement ellipsoids drawn at the 50% probability level. Intramolecular hydrogen bonds are shown as dashed lines.

**Fig. 2.**
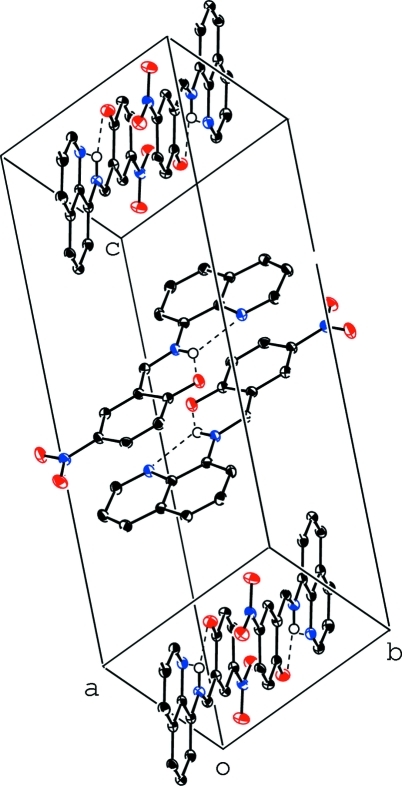
Molecular packing of (I) in the unit cell.

## supplementary crystallographic information

### Comment

In the present work, the title compound, (I), was prepared and the structure determined to explore the substituent effects of Schiff base ligands on the fluorescence of metal complexes (Shibahara *et al.*, 2010). The molecule of (I) (Fig. 1) exists in the keto form and the C=O and N–H bonds are mutually *cis*. In the structure of (I), N–H···O carbonyl and N–H···N pyridine intramolecular hydrogen bonds exist (Table 1) but there is no evidence of formal intermolecular hydrogen-bonding associations (Fig. 2).

### Experimental

8-Aminoquinoline (290.4 mg, 2.0 mmol) in toluene (14.5 ml) and 5-nitrosalicylaldehyde (333.6 mg, 1.0 mmol) in toluene (50.5 ml) were dissolved, respectively, and kept at 60 *^o^*C for one hour. The two solutions were mixed and kept at 60 *^o^*C overnight. Red brown plate-like crystals deposited: yield 438.7 mg (71%). Anal. Found (calcd for C_16_H_11_N_3_O_3_): C, 65.58(65.53); H, 3.76(3.78); N, 14.29(14.33)%.

### Refinement

The unit cell contains two crystallographically independent molecules. Trying of higher space groups, *e.g.* P2_1_/c, was not successful.

### Crystal data

**Table:**

C_16_H_11_N_3_O_3_	*Z* = 4
*M**_r_* = 293.28	*F*(000) = 608.00
Triclinic, *P*1	*D*_x_ = 1.502 Mg m^−^^3^
Hall symbol: -P 1	Mo *K*α radiation, λ = 0.71075 Å
*a* = 7.1583 (6) Å	Cell parameters from 3025 reflections
*b* = 8.2978 (7) Å	θ = 3.0–27.5°
*c* = 22.040 (2) Å	µ = 0.11 mm^−^^1^
α = 87.432 (9)°	*T* = 93 K
β = 86.258 (12)°	Block, orange
γ = 83.518 (11)°	0.25 × 0.23 × 0.18 mm
*V* = 1297.1 (2) Å^3^	

### Data collection

**Table:**

Rigaku Mercury70 CCD diffractometer	4472 reflections with *F*^2^ > 2σ(*F*^2^)
Detector resolution: 7.314 pixels mm^-1^	*R*_int_ = 0.040
ω scans	θ_max_ = 27.5°
Absorption correction: multi-scan (REQAB; Jacobson, 1998)	*h* = −9→9
*T*_min_ = 0.881, *T*_max_ = 0.981	*k* = −10→10
16456 measured reflections	*l* = −28→28
5854 independent reflections	

### Refinement

**Table:**

Refinement on *F*^2^	Secondary atom site location: difference Fourier map
*R*[*F*^2^ > 2σ(*F*^2^)] = 0.043	Hydrogen site location: inferred from neighbouring sites
*wR*(*F*^2^) = 0.130	H-atom parameters constrained
*S* = 1.06	*w* = 1/[σ^2^(*F*_o_^2^) + (0.0744*P*)^2^ + 0.0495*P*] where *P* = (*F*_o_^2^ + 2*F*_c_^2^)/3
5854 reflections	(Δ/σ)_max_ < 0.001
397 parameters	Δρ_max_ = 0.32 e Å^−^^3^
0 restraints	Δρ_min_ = −0.33 e Å^−^^3^
Primary atom site location: structure-invariant direct methods	

### Special details

**Table:**

Geometry. ENTER SPECIAL DETAILS OF THE MOLECULAR GEOMETRY
Refinement. Refinement was performed using all reflections. The weighted *R*-factor (*wR*) and goodness of fit (*S*) are based on *F*^2^. *R*-factor (gt) are based on *F*. The threshold expression of *F*^2^ > 2.0 σ(*F*^2^) is used only for calculating *R*-factor (gt).

### Fractional atomic coordinates and isotropic or equivalent isotropic displacement parameters (Å^2^)

**Table:**

	*x*	*y*	*z*	*U*_iso_*/*U*_eq_	
O(1)	0.43832 (13)	0.62852 (12)	−0.07215 (4)	0.0217 (2)	
O(2)	1.19749 (13)	0.87041 (12)	−0.17075 (4)	0.0231 (2)	
O(3)	1.17251 (13)	0.96692 (12)	−0.08065 (4)	0.0236 (2)	
O(4)	−0.16904 (14)	0.93926 (13)	0.67941 (5)	0.0267 (2)	
O(5)	−0.10119 (15)	1.04935 (12)	0.59061 (5)	0.0266 (2)	
O(6)	0.25414 (13)	0.33488 (11)	0.55531 (4)	0.0208 (2)	
N(1)	1.11180 (15)	0.89084 (13)	−0.12067 (5)	0.0169 (2)	
N(2)	0.42381 (15)	0.75275 (13)	0.03868 (5)	0.0156 (2)	
N(3)	0.09322 (15)	0.63056 (13)	0.06404 (5)	0.0170 (2)	
N(4)	−0.09901 (15)	0.93035 (14)	0.62653 (5)	0.0195 (3)	
N(5)	0.33339 (14)	0.46786 (13)	0.44950 (5)	0.0157 (2)	
N(6)	0.48679 (15)	0.16726 (13)	0.42680 (5)	0.0167 (2)	
C(1)	0.58855 (18)	0.69178 (16)	−0.08436 (6)	0.0167 (3)	
C(2)	0.69123 (19)	0.68063 (16)	−0.14301 (6)	0.0183 (3)	
C(3)	0.85732 (19)	0.74407 (16)	−0.15485 (6)	0.0180 (3)	
C(4)	0.93538 (18)	0.82506 (16)	−0.10854 (6)	0.0152 (3)	
C(5)	0.84328 (18)	0.84505 (16)	−0.05262 (6)	0.0159 (3)	
C(6)	0.67036 (18)	0.78176 (16)	−0.03944 (6)	0.0153 (3)	
C(7)	0.58246 (18)	0.80688 (16)	0.01895 (6)	0.0160 (3)	
C(8)	0.33581 (18)	0.77655 (16)	0.09739 (6)	0.0153 (3)	
C(9)	0.40980 (18)	0.85949 (16)	0.14119 (6)	0.0167 (3)	
C(10)	0.31248 (18)	0.88074 (16)	0.19822 (6)	0.0178 (3)	
C(11)	0.14418 (18)	0.81891 (16)	0.21149 (6)	0.0175 (3)	
C(12)	0.06553 (18)	0.73253 (16)	0.16752 (6)	0.0166 (3)	
C(13)	0.16067 (18)	0.71080 (15)	0.10943 (6)	0.0152 (3)	
C(14)	−0.10795 (18)	0.66561 (17)	0.17778 (6)	0.0188 (3)	
C(15)	−0.17462 (19)	0.58457 (17)	0.13279 (6)	0.0198 (3)	
C(16)	−0.06864 (18)	0.57026 (16)	0.07644 (6)	0.0176 (3)	
C(17)	0.17001 (18)	0.47021 (16)	0.57108 (6)	0.0166 (3)	
C(18)	0.07574 (19)	0.49201 (17)	0.63044 (6)	0.0188 (3)	
C(19)	−0.01010 (18)	0.63861 (17)	0.64786 (6)	0.0183 (3)	
C(20)	−0.01113 (18)	0.77500 (16)	0.60666 (6)	0.0174 (3)	
C(21)	0.07089 (18)	0.76247 (16)	0.54887 (6)	0.0174 (3)	
C(22)	0.16227 (18)	0.61281 (16)	0.52996 (6)	0.0158 (3)	
C(23)	0.24603 (18)	0.60299 (16)	0.47035 (6)	0.0168 (3)	
C(24)	0.41639 (17)	0.44225 (16)	0.39051 (6)	0.0151 (3)	
C(25)	0.41950 (18)	0.56114 (16)	0.34503 (6)	0.0173 (3)	
C(26)	0.50404 (18)	0.52268 (17)	0.28730 (6)	0.0185 (3)	
C(27)	0.58376 (18)	0.36854 (16)	0.27533 (6)	0.0176 (3)	
C(28)	0.58052 (17)	0.24344 (16)	0.32126 (6)	0.0157 (3)	
C(29)	0.49608 (17)	0.27981 (16)	0.37962 (6)	0.0149 (3)	
C(30)	0.65786 (19)	0.08012 (17)	0.31225 (6)	0.0195 (3)	
C(31)	0.64855 (19)	−0.03123 (17)	0.35941 (6)	0.0199 (3)	
C(32)	0.56198 (19)	0.01744 (16)	0.41627 (6)	0.0186 (3)	
H(2)	0.6408	0.6271	−0.1744	0.022*	
H(2N)	0.3677	0.6974	0.0135	0.019*	
H(3)	0.9211	0.7344	−0.1939	0.022*	
H(5)	0.8961	0.9018	−0.0226	0.019*	
H(5N)	0.3412	0.3835	0.4752	0.019*	
H(7)	0.6427	0.8675	0.0460	0.019*	
H(9)	0.5267	0.9024	0.1329	0.020*	
H(10)	0.3641	0.9389	0.2281	0.021*	
H(11)	0.0804	0.8342	0.2503	0.021*	
H(14)	−0.1774	0.6772	0.2158	0.023*	
H(15)	−0.2903	0.5385	0.1392	0.024*	
H(16)	−0.1171	0.5136	0.0455	0.021*	
H(18)	0.0736	0.4012	0.6582	0.023*	
H(19)	−0.0693	0.6497	0.6875	0.022*	
H(21)	0.0659	0.8548	0.5217	0.021*	
H(23)	0.2387	0.6978	0.4445	0.020*	
H(25)	0.3648	0.6686	0.3526	0.021*	
H(26)	0.5059	0.6052	0.2560	0.022*	
H(27)	0.6413	0.3454	0.2362	0.021*	
H(30)	0.7153	0.0490	0.2738	0.023*	
H(31)	0.6998	−0.1408	0.3542	0.024*	
H(32)	0.5577	−0.0619	0.4486	0.022*	

### Atomic displacement parameters (Å^2^)

**Table:**

	*U*^11^	*U*^22^	*U*^33^	*U*^12^	*U*^13^	*U*^23^
O(1)	0.0191 (5)	0.0227 (5)	0.0249 (5)	−0.0076 (4)	−0.0017 (4)	−0.0031 (4)
O(2)	0.0227 (5)	0.0284 (6)	0.0169 (5)	−0.0024 (4)	0.0083 (4)	−0.0029 (4)
O(3)	0.0219 (5)	0.0276 (6)	0.0231 (5)	−0.0097 (4)	0.0037 (4)	−0.0098 (4)
O(4)	0.0310 (6)	0.0273 (6)	0.0208 (5)	0.0010 (5)	0.0060 (4)	−0.0096 (4)
O(5)	0.0334 (6)	0.0171 (6)	0.0271 (6)	0.0043 (4)	0.0027 (4)	−0.0015 (4)
O(6)	0.0257 (5)	0.0156 (5)	0.0196 (5)	0.0030 (4)	0.0012 (4)	−0.0005 (4)
N(1)	0.0183 (6)	0.0156 (6)	0.0159 (6)	−0.0006 (5)	0.0031 (4)	−0.0018 (5)
N(2)	0.0145 (5)	0.0156 (6)	0.0168 (6)	−0.0026 (4)	0.0005 (4)	−0.0018 (4)
N(3)	0.0169 (6)	0.0146 (6)	0.0190 (6)	−0.0003 (4)	−0.0011 (4)	−0.0005 (5)
N(4)	0.0191 (6)	0.0200 (6)	0.0191 (6)	0.0004 (5)	0.0005 (5)	−0.0049 (5)
N(5)	0.0173 (6)	0.0135 (6)	0.0155 (6)	0.0008 (4)	0.0000 (4)	−0.0008 (4)
N(6)	0.0174 (6)	0.0149 (6)	0.0170 (6)	0.0002 (4)	0.0006 (4)	−0.0003 (4)
C(1)	0.0174 (7)	0.0128 (7)	0.0199 (7)	0.0001 (5)	−0.0034 (5)	−0.0001 (5)
C(2)	0.0201 (7)	0.0168 (7)	0.0186 (7)	−0.0019 (5)	−0.0044 (5)	−0.0040 (5)
C(3)	0.0229 (7)	0.0164 (7)	0.0138 (6)	0.0020 (5)	0.0000 (5)	−0.0027 (5)
C(4)	0.0150 (6)	0.0130 (7)	0.0172 (7)	−0.0009 (5)	0.0003 (5)	−0.0001 (5)
C(5)	0.0174 (7)	0.0135 (7)	0.0168 (7)	−0.0008 (5)	−0.0019 (5)	−0.0022 (5)
C(6)	0.0155 (6)	0.0135 (7)	0.0165 (7)	−0.0001 (5)	0.0002 (5)	−0.0005 (5)
C(7)	0.0165 (6)	0.0130 (7)	0.0187 (7)	−0.0012 (5)	−0.0020 (5)	−0.0012 (5)
C(8)	0.0161 (6)	0.0133 (7)	0.0156 (6)	0.0012 (5)	0.0010 (5)	0.0005 (5)
C(9)	0.0149 (6)	0.0144 (7)	0.0204 (7)	−0.0014 (5)	−0.0006 (5)	0.0008 (5)
C(10)	0.0199 (7)	0.0147 (7)	0.0190 (7)	−0.0001 (5)	−0.0032 (5)	−0.0029 (5)
C(11)	0.0190 (7)	0.0166 (7)	0.0158 (7)	0.0010 (5)	0.0022 (5)	−0.0023 (5)
C(12)	0.0164 (6)	0.0140 (7)	0.0183 (7)	0.0012 (5)	0.0003 (5)	0.0003 (5)
C(13)	0.0154 (6)	0.0118 (7)	0.0178 (7)	0.0006 (5)	−0.0012 (5)	0.0001 (5)
C(14)	0.0178 (7)	0.0200 (7)	0.0178 (7)	−0.0007 (5)	0.0040 (5)	−0.0013 (5)
C(15)	0.0150 (6)	0.0191 (7)	0.0251 (7)	−0.0024 (5)	0.0006 (5)	0.0008 (6)
C(16)	0.0175 (7)	0.0157 (7)	0.0196 (7)	−0.0005 (5)	−0.0025 (5)	−0.0003 (5)
C(17)	0.0164 (6)	0.0148 (7)	0.0187 (7)	−0.0003 (5)	−0.0031 (5)	−0.0019 (5)
C(18)	0.0208 (7)	0.0184 (7)	0.0169 (7)	−0.0010 (6)	−0.0017 (5)	0.0016 (5)
C(19)	0.0177 (7)	0.0228 (8)	0.0146 (6)	−0.0014 (5)	−0.0002 (5)	−0.0040 (5)
C(20)	0.0168 (6)	0.0152 (7)	0.0201 (7)	0.0009 (5)	−0.0013 (5)	−0.0046 (5)
C(21)	0.0178 (6)	0.0159 (7)	0.0185 (7)	−0.0004 (5)	−0.0024 (5)	−0.0007 (5)
C(22)	0.0154 (6)	0.0161 (7)	0.0160 (7)	−0.0015 (5)	−0.0006 (5)	−0.0024 (5)
C(23)	0.0164 (6)	0.0157 (7)	0.0180 (7)	−0.0001 (5)	−0.0020 (5)	−0.0006 (5)
C(24)	0.0130 (6)	0.0172 (7)	0.0152 (6)	−0.0012 (5)	−0.0005 (5)	−0.0020 (5)
C(25)	0.0159 (6)	0.0139 (7)	0.0216 (7)	0.0010 (5)	−0.0005 (5)	−0.0013 (5)
C(26)	0.0184 (7)	0.0190 (7)	0.0174 (7)	−0.0012 (5)	−0.0006 (5)	0.0040 (5)
C(27)	0.0169 (6)	0.0208 (7)	0.0146 (6)	−0.0007 (5)	0.0011 (5)	−0.0006 (5)
C(28)	0.0134 (6)	0.0173 (7)	0.0164 (7)	−0.0010 (5)	−0.0010 (5)	−0.0021 (5)
C(29)	0.0130 (6)	0.0161 (7)	0.0158 (6)	−0.0021 (5)	−0.0011 (5)	−0.0016 (5)
C(30)	0.0207 (7)	0.0212 (7)	0.0158 (7)	0.0008 (6)	0.0014 (5)	−0.0039 (5)
C(31)	0.0232 (7)	0.0151 (7)	0.0207 (7)	0.0016 (5)	0.0007 (5)	−0.0040 (5)
C(32)	0.0212 (7)	0.0147 (7)	0.0191 (7)	−0.0004 (5)	0.0010 (5)	0.0011 (5)

### Geometric parameters (Å, °)

**Table:**

O(1)—C(1)	1.2576 (17)	C(19)—C(20)	1.4186 (19)
O(2)—N(1)	1.2362 (14)	C(20)—C(21)	1.3705 (18)
O(3)—N(1)	1.2358 (15)	C(21)—C(22)	1.4040 (18)
O(4)—N(4)	1.2402 (15)	C(22)—C(23)	1.4100 (18)
O(5)—N(4)	1.2370 (15)	C(24)—C(25)	1.3755 (18)
O(6)—C(17)	1.2647 (16)	C(24)—C(29)	1.4274 (18)
N(1)—C(4)	1.4362 (17)	C(25)—C(26)	1.4069 (18)
N(2)—C(7)	1.3091 (17)	C(26)—C(27)	1.3700 (19)
N(2)—C(8)	1.4137 (17)	C(27)—C(28)	1.4181 (18)
N(3)—C(13)	1.3684 (18)	C(28)—C(29)	1.4153 (18)
N(3)—C(16)	1.3209 (17)	C(28)—C(30)	1.4223 (19)
N(4)—C(20)	1.4425 (17)	C(30)—C(31)	1.3628 (19)
N(5)—C(23)	1.3076 (16)	C(31)—C(32)	1.4160 (18)
N(5)—C(24)	1.4085 (17)	N(2)—H(2N)	0.880
N(6)—C(29)	1.3696 (17)	N(5)—H(5N)	0.880
N(6)—C(32)	1.3219 (17)	C(2)—H(2)	0.950
C(1)—C(2)	1.4461 (18)	C(3)—H(3)	0.950
C(1)—C(6)	1.4564 (20)	C(5)—H(5)	0.950
C(2)—C(3)	1.3591 (20)	C(7)—H(7)	0.950
C(3)—C(4)	1.4235 (20)	C(9)—H(9)	0.950
C(4)—C(5)	1.3670 (18)	C(10)—H(10)	0.950
C(5)—C(6)	1.4066 (19)	C(11)—H(11)	0.950
C(6)—C(7)	1.4086 (18)	C(14)—H(14)	0.950
C(8)—C(9)	1.3765 (20)	C(15)—H(15)	0.950
C(8)—C(13)	1.4275 (19)	C(16)—H(16)	0.950
C(9)—C(10)	1.4050 (18)	C(18)—H(18)	0.950
C(10)—C(11)	1.3712 (19)	C(19)—H(19)	0.950
C(11)—C(12)	1.4122 (20)	C(21)—H(21)	0.950
C(12)—C(13)	1.4194 (18)	C(23)—H(23)	0.950
C(12)—C(14)	1.4175 (19)	C(25)—H(25)	0.950
C(14)—C(15)	1.363 (2)	C(26)—H(26)	0.950
C(15)—C(16)	1.4149 (18)	C(27)—H(27)	0.950
C(17)—C(18)	1.4416 (18)	C(30)—H(30)	0.950
C(17)—C(22)	1.4561 (18)	C(31)—H(31)	0.950
C(18)—C(19)	1.3591 (19)	C(32)—H(32)	0.950
			
O(1)···N(2)	2.6849 (15)	H(30)···H(31)	2.3218
O(1)···C(7)	2.8580 (17)	H(31)···H(32)	2.3434
O(2)···C(3)	2.7538 (17)	O(1)···H(2N)^ii^	3.1599
O(2)···C(5)	3.5301 (16)	O(1)···H(15)^iii^	2.4380
O(3)···C(3)	3.5887 (18)	O(1)···H(16)^iii^	2.7199
O(3)···C(5)	2.6927 (17)	O(2)···H(2)^iv^	3.5621
O(4)···C(19)	2.7227 (17)	O(2)···H(9)^vii^	3.0570
O(4)···C(21)	3.5417 (17)	O(2)···H(11)^v^	3.4655
O(5)···C(19)	3.5852 (17)	O(2)···H(15)^ii^	3.4319
O(5)···C(21)	2.7233 (16)	O(2)···H(27)^vi^	2.4828
O(6)···N(5)	2.5915 (14)	O(2)···H(30)^vi^	2.4025
O(6)···N(6)	3.4561 (14)	O(3)···H(2N)^iv^	3.2430
O(6)···C(23)	2.8409 (16)	O(3)···H(5)^vii^	2.5621
N(2)···N(3)	2.6942 (16)	O(3)···H(7)^vii^	2.2044
N(2)···C(1)	2.9277 (17)	O(3)···H(9)^vii^	2.6883
N(3)···C(14)	2.8182 (17)	O(4)···H(3)^xii^	3.2655
N(5)···N(6)	2.6645 (15)	O(4)···H(10)^ix^	2.5738
N(5)···C(17)	2.8529 (17)	O(4)···H(11)^ix^	2.6445
N(6)···C(30)	2.8207 (17)	O(4)···H(25)^ix^	3.4554
C(1)···C(4)	2.8400 (19)	O(4)···H(30)^x^	3.4840
C(2)···C(5)	2.807 (2)	O(5)···H(21)^ix^	2.5710
C(3)···C(6)	2.8092 (19)	O(5)···H(23)^ix^	2.3356
C(7)···C(9)	2.9199 (19)	O(5)···H(25)^ix^	3.0924
C(8)···C(11)	2.8017 (18)	O(5)···H(31)^x^	3.3681
C(8)···C(16)	3.5902 (20)	O(5)···H(32)^viii^	3.4236
C(9)···C(12)	2.8044 (19)	O(6)···H(21)^viii^	3.4880
C(10)···C(13)	2.8053 (20)	O(6)···H(23)^viii^	3.5688
C(12)···C(16)	2.7479 (20)	O(6)···H(25)^x^	3.5018
C(13)···C(15)	2.7351 (20)	O(6)···H(31)^xi^	2.5222
C(17)···C(20)	2.8279 (18)	O(6)···H(32)^xi^	2.5031
C(18)···C(21)	2.8105 (19)	N(1)···H(7)^vii^	3.3602
C(19)···C(22)	2.8093 (18)	N(1)···H(9)^vii^	3.2529
C(23)···C(25)	2.9719 (19)	N(1)···H(27)^vi^	3.5387
C(24)···C(27)	2.7983 (18)	N(1)···H(30)^vi^	3.5525
C(24)···C(32)	3.5920 (19)	N(2)···H(5)^v^	3.4815
C(25)···C(28)	2.8127 (18)	N(2)···H(7)^v^	3.5900
C(26)···C(29)	2.8014 (19)	N(2)···H(15)^iv^	3.4162
C(28)···C(32)	2.7545 (19)	N(3)···H(5)^i^	3.1548
C(29)···C(31)	2.7303 (19)	N(3)···H(16)^iii^	2.7290
O(1)···O(2)^i^	3.3227 (13)	N(4)···H(11)^ix^	3.4326
O(1)···O(3)^i^	3.2146 (14)	N(4)···H(23)^ix^	3.4701
O(1)···N(1)^i^	3.2115 (14)	N(4)···H(31)^x^	3.5704
O(1)···N(2)^ii^	3.2723 (15)	N(5)···H(5N)^x^	3.3226
O(1)···C(15)^iii^	3.1258 (18)	N(6)···H(21)^x^	3.4494
O(1)···C(16)^iii^	3.2809 (17)	N(6)···H(32)^xi^	2.8528
O(2)···O(1)^iv^	3.3227 (13)	C(1)···H(2N)^ii^	3.5140
O(2)···C(11)^v^	3.4770 (16)	C(1)···H(9)^v^	3.5112
O(2)···C(27)^vi^	3.3066 (16)	C(1)···H(15)^iii^	3.3366
O(2)···C(30)^vi^	3.2444 (16)	C(2)···H(10)^v^	3.5949
O(3)···O(1)^iv^	3.2146 (14)	C(2)···H(15)^iii^	3.5615
O(3)···N(2)^iv^	3.5761 (15)	C(2)···H(27)^ii^	3.2746
O(3)···C(1)^iv^	3.5409 (16)	C(3)···H(10)^v^	3.3240
O(3)···C(5)^vii^	3.3767 (17)	C(3)···H(19)^xiii^	3.5875
O(3)···C(7)^vii^	3.1196 (18)	C(4)···H(16)^ii^	3.2525
O(3)···C(12)^v^	3.4391 (16)	C(5)···H(5)^vii^	3.4991
O(3)···C(13)^v^	3.4420 (16)	C(5)···H(16)^iv^	3.4195
O(4)···N(6)^viii^	3.5733 (17)	C(5)···H(16)^ii^	3.3767
O(4)···C(10)^ix^	3.1957 (17)	C(6)···H(7)^v^	3.4706
O(4)···C(11)^ix^	3.2263 (18)	C(6)···H(9)^v^	3.4871
O(4)···C(28)^viii^	3.4627 (17)	C(6)···H(16)^iv^	3.1633
O(4)···C(29)^viii^	3.4710 (18)	C(7)···H(7)^v^	3.3041
O(4)···C(30)^viii^	3.5134 (17)	C(7)···H(15)^iv^	3.4837
O(4)···C(31)^viii^	3.5819 (17)	C(7)···H(16)^iv^	3.1220
O(5)···N(6)^viii^	3.5096 (16)	C(8)···H(5)^v^	3.3999
O(5)···C(21)^ix^	3.3904 (17)	C(8)···H(15)^iv^	3.2983
O(5)···C(23)^ix^	3.2189 (16)	C(9)···H(15)^iv^	3.2270
O(5)···C(31)^x^	3.4750 (18)	C(9)···H(26)	3.2792
O(5)···C(32)^viii^	3.4199 (18)	C(10)···H(3)^v^	3.4339
O(6)···N(5)^x^	3.5275 (15)	C(10)···H(26)	2.8325
O(6)···C(21)^viii^	3.5533 (17)	C(11)···H(26)	3.1547
O(6)···C(24)^x^	3.4581 (17)	C(12)···H(2)^ii^	3.4540
O(6)···C(25)^x^	3.5102 (17)	C(13)···H(2)^ii^	3.3068
O(6)···C(31)^xi^	3.1172 (16)	C(14)···H(3)^ii^	3.4476
O(6)···C(32)^xi^	3.1219 (16)	C(14)···H(9)^i^	3.2684
N(1)···O(1)^iv^	3.2115 (14)	C(14)···H(26)^i^	3.2372
N(1)···C(11)^v^	3.4950 (17)	C(14)···H(27)^i^	3.5220
N(1)···C(12)^v^	3.3809 (17)	C(15)···H(3)^ii^	3.3208
N(2)···O(1)^ii^	3.2723 (15)	C(15)···H(7)^i^	3.1802
N(2)···O(3)^i^	3.5761 (15)	C(15)···H(9)^i^	3.2019
N(3)···C(1)^ii^	3.3457 (17)	C(15)···H(26)^i^	3.4320
N(3)···C(2)^ii^	3.3356 (17)	C(15)···H(27)^i^	3.2646
N(3)···C(5)^i^	3.5286 (17)	C(16)···H(5)^i^	3.4314
N(4)···N(6)^viii^	3.2805 (17)	C(16)···H(7)^i^	3.1122
N(4)···C(29)^viii^	3.5113 (18)	C(16)···H(16)^iii^	2.9944
N(4)···C(30)^x^	3.5049 (18)	C(17)···H(23)^viii^	3.4232
N(4)···C(31)^x^	3.4579 (18)	C(17)···H(31)^xi^	3.2059
N(4)···C(32)^viii^	3.4796 (18)	C(18)···H(23)^viii^	3.4341
N(5)···O(6)^x^	3.5275 (15)	C(18)···H(25)^viii^	3.5517
N(5)···N(5)^x^	3.4664 (15)	C(18)···H(31)^xi^	3.1807
N(5)···C(18)^viii^	3.4918 (18)	C(20)···H(5N)^viii^	3.4693
N(5)···C(19)^viii^	3.4670 (18)	C(21)···H(5N)^viii^	3.3937
N(5)···C(20)^viii^	3.5441 (18)	C(21)···H(21)^ix^	3.5381
N(5)···C(23)^x^	3.5681 (17)	C(22)···H(5N)^x^	3.5524
N(6)···O(4)^viii^	3.5733 (17)	C(23)···H(5N)^x^	3.2762
N(6)···O(5)^viii^	3.5096 (16)	C(24)···H(19)^viii^	3.2891
N(6)···N(4)^viii^	3.2805 (17)	C(25)···H(18)^viii^	3.5130
N(6)···C(20)^viii^	3.5064 (17)	C(25)···H(19)^viii^	3.3464
N(6)···C(21)^x^	3.3644 (18)	C(25)···H(31)^xiv^	3.3768
N(6)···C(22)^x^	3.4717 (18)	C(26)···H(2)^ii^	3.1170
C(1)···O(3)^i^	3.5409 (16)	C(26)···H(14)^iv^	3.0551
C(1)···N(3)^ii^	3.3457 (17)	C(26)···H(15)^iv^	3.4964
C(2)···N(3)^ii^	3.3356 (17)	C(26)···H(18)^x^	3.4580
C(2)···C(13)^ii^	3.3661 (18)	C(26)···H(19)^viii^	3.5704
C(2)···C(16)^ii^	3.5720 (19)	C(27)···H(2)^ii^	2.8250
C(3)···C(10)^v^	3.3338 (18)	C(27)···H(14)^iv^	3.4053
C(3)···C(15)^ii^	3.3851 (19)	C(27)···H(15)^iv^	3.3712
C(3)···C(16)^ii^	3.3353 (18)	C(29)···H(19)^viii^	3.4606
C(4)···C(9)^v^	3.4753 (18)	C(30)···H(10)^xv^	3.2400
C(4)···C(10)^v^	3.4730 (18)	C(31)···H(18)^xi^	3.4871
C(4)···C(16)^ii^	3.3681 (18)	C(31)···H(25)^xv^	3.4007
C(5)···O(3)^vii^	3.3767 (17)	C(32)···H(21)^x^	3.3663
C(5)···N(3)^iv^	3.5286 (17)	C(32)···H(32)^xi^	3.0711
C(5)···C(8)^v^	3.3845 (18)	H(2)···O(2)^i^	3.5621
C(5)···C(9)^v^	3.4833 (18)	H(2)···C(12)^ii^	3.4540
C(6)···C(16)^iv^	3.5460 (19)	H(2)···C(13)^ii^	3.3068
C(7)···O(3)^vii^	3.1196 (18)	H(2)···C(26)^ii^	3.1170
C(7)···C(7)^v^	3.3760 (19)	H(2)···C(27)^ii^	2.8250
C(7)···C(15)^iv^	3.4860 (19)	H(2)···H(15)^iii^	3.0355
C(7)···C(16)^iv^	3.2835 (18)	H(2)···H(19)^xiii^	3.5800
C(8)···C(5)^v^	3.3845 (18)	H(2)···H(26)^ii^	3.0110
C(9)···C(4)^v^	3.4753 (18)	H(2)···H(27)^ii^	2.4923
C(9)···C(5)^v^	3.4833 (18)	H(2N)···O(1)^ii^	3.1599
C(9)···C(15)^iv^	3.5386 (18)	H(2N)···O(3)^i^	3.2430
C(10)···O(4)^ix^	3.1957 (17)	H(2N)···C(1)^ii^	3.5140
C(10)···C(3)^v^	3.3338 (18)	H(2N)···H(16)^iii^	3.0316
C(10)···C(4)^v^	3.4730 (18)	H(3)···O(4)^xiii^	3.2655
C(11)···O(2)^v^	3.4770 (16)	H(3)···C(10)^v^	3.4339
C(11)···O(4)^ix^	3.2263 (18)	H(3)···C(14)^ii^	3.4476
C(11)···N(1)^v^	3.4950 (17)	H(3)···C(15)^ii^	3.3208
C(12)···O(3)^v^	3.4391 (16)	H(3)···H(10)^v^	3.2944
C(12)···N(1)^v^	3.3809 (17)	H(3)···H(15)^ii^	3.5195
C(13)···O(3)^v^	3.4420 (16)	H(3)···H(19)^xiii^	2.7307
C(13)···C(2)^ii^	3.3661 (18)	H(3)···H(27)^vi^	3.2127
C(15)···O(1)^iii^	3.1258 (18)	H(5)···O(3)^vii^	2.5621
C(15)···C(3)^ii^	3.3851 (19)	H(5)···N(2)^v^	3.4815
C(15)···C(7)^i^	3.4860 (19)	H(5)···N(3)^iv^	3.1548
C(15)···C(9)^i^	3.5386 (18)	H(5)···C(5)^vii^	3.4991
C(16)···O(1)^iii^	3.2809 (17)	H(5)···C(8)^v^	3.3999
C(16)···C(2)^ii^	3.5720 (19)	H(5)···C(16)^iv^	3.4314
C(16)···C(3)^ii^	3.3353 (18)	H(5)···H(5)^vii^	2.5980
C(16)···C(4)^ii^	3.3681 (18)	H(5)···H(16)^iv^	3.5008
C(16)···C(6)^i^	3.5460 (19)	H(5N)···N(5)^x^	3.3226
C(16)···C(7)^i^	3.2835 (18)	H(5N)···C(20)^viii^	3.4693
C(17)···C(22)^viii^	3.5057 (20)	H(5N)···C(21)^viii^	3.3937
C(17)···C(23)^viii^	3.2993 (19)	H(5N)···C(22)^x^	3.5524
C(17)···C(24)^x^	3.2981 (19)	H(5N)···C(23)^x^	3.2762
C(17)···C(25)^x^	3.5516 (19)	H(5N)···H(5N)^x^	3.4077
C(17)···C(29)^x^	3.5856 (20)	H(5N)···H(23)^x^	3.5725
C(18)···N(5)^viii^	3.4918 (18)	H(5N)···H(32)^xi^	3.1277
C(18)···C(23)^viii^	3.478 (2)	H(7)···O(3)^vii^	2.2044
C(18)···C(24)^viii^	3.5605 (19)	H(7)···N(1)^vii^	3.3602
C(18)···C(26)^x^	3.5999 (20)	H(7)···N(2)^v^	3.5900
C(19)···N(5)^viii^	3.4670 (18)	H(7)···C(6)^v^	3.4706
C(19)···C(24)^viii^	3.2305 (19)	H(7)···C(7)^v^	3.3041
C(19)···C(25)^viii^	3.5176 (20)	H(7)···C(15)^iv^	3.1802
C(19)···C(27)^x^	3.5788 (19)	H(7)···C(16)^iv^	3.1122
C(19)···C(28)^x^	3.4481 (19)	H(7)···H(7)^v^	3.4988
C(19)···C(29)^viii^	3.5544 (18)	H(7)···H(15)^iv^	3.3512
C(20)···N(5)^viii^	3.5441 (18)	H(7)···H(16)^iv^	3.2314
C(20)···N(6)^viii^	3.5064 (17)	H(9)···O(2)^vii^	3.0570
C(20)···C(24)^viii^	3.5800 (19)	H(9)···O(3)^vii^	2.6883
C(20)···C(28)^x^	3.5439 (19)	H(9)···N(1)^vii^	3.2529
C(20)···C(29)^viii^	3.5435 (18)	H(9)···C(1)^v^	3.5112
C(20)···C(30)^x^	3.536 (2)	H(9)···C(6)^v^	3.4871
C(21)···O(5)^ix^	3.3904 (17)	H(9)···C(14)^iv^	3.2684
C(21)···O(6)^viii^	3.5533 (17)	H(9)···C(15)^iv^	3.2019
C(21)···N(6)^x^	3.3644 (18)	H(9)···H(14)^iv^	3.2600
C(21)···C(29)^x^	3.5438 (19)	H(9)···H(15)^iv^	3.1538
C(21)···C(32)^x^	3.508 (2)	H(9)···H(26)	3.5922
C(22)···N(6)^x^	3.4717 (18)	H(10)···O(4)^ix^	2.5738
C(22)···C(17)^viii^	3.5057 (20)	H(10)···C(2)^v^	3.5949
C(22)···C(22)^viii^	3.5000 (19)	H(10)···C(3)^v^	3.3240
C(22)···C(23)^viii^	3.5964 (20)	H(10)···C(30)^xiv^	3.2400
C(22)···C(24)^x^	3.5616 (19)	H(10)···H(3)^v^	3.2944
C(22)···C(29)^x^	3.4690 (20)	H(10)···H(25)	3.4647
C(23)···O(5)^ix^	3.2189 (16)	H(10)···H(26)	2.8935
C(23)···N(5)^x^	3.5681 (17)	H(10)···H(30)^xiv^	3.0175
C(23)···C(17)^viii^	3.2993 (19)	H(11)···O(2)^v^	3.4655
C(23)···C(18)^viii^	3.478 (2)	H(11)···O(4)^ix^	2.6445
C(23)···C(22)^viii^	3.5964 (20)	H(11)···N(4)^ix^	3.4326
C(24)···O(6)^x^	3.4581 (17)	H(11)···H(18)^viii^	2.9897
C(24)···C(17)^x^	3.2981 (19)	H(11)···H(25)	3.2864
C(24)···C(18)^viii^	3.5605 (19)	H(11)···H(26)	3.4097
C(24)···C(19)^viii^	3.2305 (19)	H(11)···H(30)^xvi^	3.0218
C(24)···C(20)^viii^	3.5800 (19)	H(11)···H(31)^xvi^	3.4360
C(24)···C(22)^x^	3.5616 (19)	H(14)···C(26)^i^	3.0551
C(25)···O(6)^x^	3.5102 (17)	H(14)···C(27)^i^	3.4053
C(25)···C(17)^x^	3.5516 (19)	H(14)···H(9)^i^	3.2600
C(25)···C(19)^viii^	3.5176 (20)	H(14)···H(18)^viii^	2.9462
C(26)···C(18)^x^	3.5999 (20)	H(14)···H(26)^i^	2.5054
C(27)···O(2)^vi^	3.3066 (16)	H(14)···H(27)^i^	3.1754
C(27)···C(19)^x^	3.5788 (19)	H(14)···H(30)^xvi^	3.3813
C(28)···O(4)^viii^	3.4627 (17)	H(14)···H(31)^xvi^	3.4801
C(28)···C(19)^x^	3.4481 (19)	H(15)···O(1)^iii^	2.4380
C(28)···C(20)^x^	3.5439 (19)	H(15)···O(2)^ii^	3.4319
C(29)···O(4)^viii^	3.4710 (18)	H(15)···N(2)^i^	3.4162
C(29)···N(4)^viii^	3.5113 (18)	H(15)···C(1)^iii^	3.3366
C(29)···C(17)^x^	3.5856 (20)	H(15)···C(2)^iii^	3.5615
C(29)···C(19)^viii^	3.5544 (18)	H(15)···C(7)^i^	3.4837
C(29)···C(20)^viii^	3.5435 (18)	H(15)···C(8)^i^	3.2983
C(29)···C(21)^x^	3.5438 (19)	H(15)···C(9)^i^	3.2270
C(29)···C(22)^x^	3.4690 (20)	H(15)···C(26)^i^	3.4964
C(30)···O(2)^vi^	3.2444 (16)	H(15)···C(27)^i^	3.3712
C(30)···O(4)^viii^	3.5134 (17)	H(15)···H(2)^iii^	3.0355
C(30)···N(4)^x^	3.5049 (18)	H(15)···H(3)^ii^	3.5195
C(30)···C(20)^x^	3.536 (2)	H(15)···H(7)^i^	3.3512
C(31)···O(4)^viii^	3.5819 (17)	H(15)···H(9)^i^	3.1538
C(31)···O(5)^x^	3.4750 (18)	H(15)···H(26)^i^	2.9198
C(31)···O(6)^xi^	3.1172 (16)	H(15)···H(27)^i^	2.6667
C(31)···N(4)^x^	3.4579 (18)	H(16)···O(1)^iii^	2.7199
C(32)···O(5)^viii^	3.4199 (18)	H(16)···N(3)^iii^	2.7290
C(32)···O(6)^xi^	3.1219 (16)	H(16)···C(4)^ii^	3.2525
C(32)···N(4)^viii^	3.4796 (18)	H(16)···C(5)^i^	3.4195
C(32)···C(21)^x^	3.508 (2)	H(16)···C(5)^ii^	3.3767
O(1)···H(2)	2.5982	H(16)···C(6)^i^	3.1633
O(1)···H(2N)	2.0128	H(16)···C(7)^i^	3.1220
O(2)···H(3)	2.4770	H(16)···C(16)^iii^	2.9944
O(3)···H(5)	2.3857	H(16)···H(2N)^iii^	3.0316
O(4)···H(19)	2.4301	H(16)···H(5)^i^	3.5008
O(5)···H(21)	2.4272	H(16)···H(7)^i^	3.2314
O(6)···H(5N)	1.8755	H(16)···H(16)^iii^	2.5294
O(6)···H(18)	2.5855	H(18)···C(25)^viii^	3.5130
N(1)···H(3)	2.6513	H(18)···C(26)^x^	3.4580
N(1)···H(5)	2.5714	H(18)···C(31)^xi^	3.4871
N(2)···H(9)	2.6515	H(18)···H(11)^viii^	2.9897
N(3)···H(2N)	2.3052	H(18)···H(14)^viii^	2.9462
N(3)···H(15)	3.2598	H(18)···H(23)^viii^	3.4689
N(4)···H(19)	2.6307	H(18)···H(25)^viii^	3.2803
N(4)···H(21)	2.5948	H(18)···H(31)^xi^	2.5653
N(5)···H(25)	2.6635	H(19)···C(3)^xii^	3.5875
N(6)···H(5N)	2.2464	H(19)···C(24)^viii^	3.2891
N(6)···H(31)	3.2539	H(19)···C(25)^viii^	3.3464
C(1)···H(2N)	2.5884	H(19)···C(26)^viii^	3.5704
C(1)···H(3)	3.3144	H(19)···C(29)^viii^	3.4606
C(1)···H(5)	3.3474	H(19)···H(2)^xii^	3.5800
C(1)···H(7)	3.3429	H(19)···H(3)^xii^	2.7307
C(3)···H(5)	3.2882	H(19)···H(27)^x^	3.5945
C(4)···H(2)	3.2632	H(21)···O(5)^ix^	2.5710
C(5)···H(3)	3.2810	H(21)···O(6)^viii^	3.4880
C(5)···H(7)	2.5273	H(21)···N(6)^x^	3.4494
C(6)···H(2)	3.3204	H(21)···C(21)^ix^	3.5381
C(6)···H(2N)	2.5465	H(21)···C(32)^x^	3.3663
C(7)···H(5)	2.5626	H(21)···H(21)^ix^	2.6477
C(7)···H(9)	2.6583	H(21)···H(32)^x^	3.4667
C(8)···H(7)	2.5790	H(23)···O(5)^ix^	2.3356
C(8)···H(10)	3.2614	H(23)···O(6)^viii^	3.5688
C(9)···H(2N)	3.2193	H(23)···N(4)^ix^	3.4701
C(9)···H(7)	2.5968	H(23)···C(17)^viii^	3.4232
C(9)···H(11)	3.2734	H(23)···C(18)^viii^	3.4341
C(11)···H(9)	3.2686	H(23)···H(5N)^x^	3.5725
C(11)···H(14)	2.6951	H(23)···H(18)^viii^	3.4689
C(12)···H(10)	3.2687	H(23)···H(32)^xiv^	3.2042
C(12)···H(15)	3.2701	H(25)···O(4)^ix^	3.4554
C(13)···H(2N)	2.5009	H(25)···O(5)^ix^	3.0924
C(13)···H(9)	3.2942	H(25)···O(6)^x^	3.5018
C(13)···H(11)	3.3076	H(25)···C(18)^viii^	3.5517
C(13)···H(14)	3.2847	H(25)···C(31)^xiv^	3.4007
C(13)···H(16)	3.1500	H(25)···H(10)	3.4647
C(14)···H(11)	2.6905	H(25)···H(11)	3.2864
C(14)···H(16)	3.2355	H(25)···H(18)^viii^	3.2803
C(16)···H(2N)	3.5833	H(25)···H(31)^xiv^	3.0205
C(16)···H(14)	3.2574	H(25)···H(32)^xiv^	3.5902
C(17)···H(5N)	2.4667	H(26)···C(9)	3.2792
C(17)···H(19)	3.3064	H(26)···C(10)	2.8325
C(17)···H(21)	3.3438	H(26)···C(11)	3.1547
C(17)···H(23)	3.3402	H(26)···C(14)^iv^	3.2372
C(19)···H(21)	3.2897	H(26)···C(15)^iv^	3.4320
C(20)···H(18)	3.2600	H(26)···H(2)^ii^	3.0110
C(21)···H(19)	3.2829	H(26)···H(9)	3.5922
C(21)···H(23)	2.5753	H(26)···H(10)	2.8935
C(22)···H(5N)	2.4845	H(26)···H(11)	3.4097
C(22)···H(18)	3.3238	H(26)···H(14)^iv^	2.5054
C(23)···H(21)	2.5925	H(26)···H(15)^iv^	2.9198
C(23)···H(25)	2.7294	H(26)···H(31)^xiv^	3.5373
C(24)···H(23)	2.6359	H(27)···O(2)^vi^	2.4828
C(24)···H(26)	3.2577	H(27)···N(1)^vi^	3.5387
C(25)···H(5N)	3.2120	H(27)···C(2)^ii^	3.2746
C(25)···H(23)	2.6988	H(27)···C(14)^iv^	3.5220
C(25)···H(27)	3.2756	H(27)···C(15)^iv^	3.2646
C(27)···H(25)	3.2719	H(27)···H(2)^ii^	2.4923
C(27)···H(30)	2.7123	H(27)···H(3)^vi^	3.2127
C(28)···H(26)	3.2731	H(27)···H(14)^iv^	3.1754
C(28)···H(31)	3.2677	H(27)···H(15)^iv^	2.6667
C(29)···H(5N)	2.4594	H(27)···H(19)^x^	3.5945
C(29)···H(25)	3.2962	H(30)···O(2)^vi^	2.4025
C(29)···H(27)	3.3045	H(30)···O(4)^x^	3.4840
C(29)···H(30)	3.2877	H(30)···N(1)^vi^	3.5525
C(29)···H(32)	3.1569	H(30)···H(10)^xv^	3.0175
C(30)···H(27)	2.7034	H(30)···H(11)^xvii^	3.0218
C(30)···H(32)	3.2448	H(30)···H(14)^xvii^	3.3813
C(32)···H(5N)	3.5228	H(31)···O(5)^x^	3.3681
C(32)···H(30)	3.2663	H(31)···O(6)^xi^	2.5222
H(2)···H(3)	2.2939	H(31)···N(4)^x^	3.5704
H(2N)···H(7)	2.6984	H(31)···C(17)^xi^	3.2059
H(2N)···H(9)	3.5131	H(31)···C(18)^xi^	3.1807
H(5)···H(7)	2.3191	H(31)···C(25)^xv^	3.3768
H(5N)···H(23)	2.6976	H(31)···H(11)^xvii^	3.4360
H(5N)···H(25)	3.5203	H(31)···H(14)^xvii^	3.4801
H(7)···H(9)	2.0562	H(31)···H(18)^xi^	2.5653
H(9)···H(10)	2.3471	H(31)···H(25)^xv^	3.0205
H(10)···H(11)	2.3129	H(31)···H(26)^xv^	3.5373
H(11)···H(14)	2.5485	H(32)···O(5)^viii^	3.4236
H(14)···H(15)	2.3232	H(32)···O(6)^xi^	2.5031
H(15)···H(16)	2.3424	H(32)···N(6)^xi^	2.8528
H(18)···H(19)	2.2961	H(32)···C(32)^xi^	3.0711
H(21)···H(23)	2.3843	H(32)···H(5N)^xi^	3.1277
H(23)···H(25)	2.1751	H(32)···H(21)^x^	3.4667
H(25)···H(26)	2.3504	H(32)···H(23)^xv^	3.2042
H(26)···H(27)	2.3097	H(32)···H(25)^xv^	3.5902
H(27)···H(30)	2.5687	H(32)···H(32)^xi^	2.5768
			
O(2)—N(1)—O(3)	122.13 (11)	C(25)—C(26)—C(27)	121.26 (12)
O(2)—N(1)—C(4)	119.13 (11)	C(26)—C(27)—C(28)	120.16 (12)
O(3)—N(1)—C(4)	118.74 (10)	C(27)—C(28)—C(29)	119.39 (12)
C(7)—N(2)—C(8)	124.94 (12)	C(27)—C(28)—C(30)	123.60 (12)
C(13)—N(3)—C(16)	116.79 (11)	C(29)—C(28)—C(30)	117.01 (12)
O(4)—N(4)—O(5)	122.50 (11)	N(6)—C(29)—C(24)	117.68 (11)
O(4)—N(4)—C(20)	118.35 (11)	N(6)—C(29)—C(28)	123.54 (12)
O(5)—N(4)—C(20)	119.16 (11)	C(24)—C(29)—C(28)	118.78 (12)
C(23)—N(5)—C(24)	127.29 (11)	C(28)—C(30)—C(31)	119.17 (12)
C(29)—N(6)—C(32)	117.14 (11)	C(30)—C(31)—C(32)	119.55 (12)
O(1)—C(1)—C(2)	122.95 (13)	N(6)—C(32)—C(31)	123.59 (12)
O(1)—C(1)—C(6)	121.39 (12)	C(7)—N(2)—H(2N)	117.526
C(2)—C(1)—C(6)	115.65 (12)	C(8)—N(2)—H(2N)	117.532
C(1)—C(2)—C(3)	122.35 (13)	C(23)—N(5)—H(5N)	116.360
C(2)—C(3)—C(4)	119.79 (12)	C(24)—N(5)—H(5N)	116.349
N(1)—C(4)—C(3)	119.96 (11)	C(1)—C(2)—H(2)	118.830
N(1)—C(4)—C(5)	118.85 (12)	C(3)—C(2)—H(2)	118.824
C(3)—C(4)—C(5)	121.17 (12)	C(2)—C(3)—H(3)	120.110
C(4)—C(5)—C(6)	120.09 (13)	C(4)—C(3)—H(3)	120.105
C(1)—C(6)—C(5)	120.86 (12)	C(4)—C(5)—H(5)	119.957
C(1)—C(6)—C(7)	121.86 (12)	C(6)—C(5)—H(5)	119.955
C(5)—C(6)—C(7)	117.27 (13)	N(2)—C(7)—H(7)	117.631
N(2)—C(7)—C(6)	124.73 (13)	C(6)—C(7)—H(7)	117.636
N(2)—C(8)—C(9)	123.28 (12)	C(8)—C(9)—H(9)	119.987
N(2)—C(8)—C(13)	116.17 (12)	C(10)—C(9)—H(9)	119.974
C(9)—C(8)—C(13)	120.54 (12)	C(9)—C(10)—H(10)	119.473
C(8)—C(9)—C(10)	120.04 (12)	C(11)—C(10)—H(10)	119.465
C(9)—C(10)—C(11)	121.06 (13)	C(10)—C(11)—H(11)	119.951
C(10)—C(11)—C(12)	120.09 (12)	C(12)—C(11)—H(11)	119.955
C(11)—C(12)—C(13)	119.79 (12)	C(12)—C(14)—H(14)	120.147
C(11)—C(12)—C(14)	123.36 (12)	C(15)—C(14)—H(14)	120.151
C(13)—C(12)—C(14)	116.85 (12)	C(14)—C(15)—H(15)	120.576
N(3)—C(13)—C(8)	118.00 (11)	C(16)—C(15)—H(15)	120.575
N(3)—C(13)—C(12)	123.53 (12)	N(3)—C(16)—H(16)	117.860
C(8)—C(13)—C(12)	118.47 (12)	C(15)—C(16)—H(16)	117.867
C(12)—C(14)—C(15)	119.70 (12)	C(17)—C(18)—H(18)	119.021
C(14)—C(15)—C(16)	118.85 (13)	C(19)—C(18)—H(18)	119.027
N(3)—C(16)—C(15)	124.27 (13)	C(18)—C(19)—H(19)	120.077
O(6)—C(17)—C(18)	122.08 (12)	C(20)—C(19)—H(19)	120.083
O(6)—C(17)—C(22)	121.67 (11)	C(20)—C(21)—H(21)	120.090
C(18)—C(17)—C(22)	116.25 (11)	C(22)—C(21)—H(21)	120.102
C(17)—C(18)—C(19)	121.95 (12)	N(5)—C(23)—H(23)	118.877
C(18)—C(19)—C(20)	119.84 (12)	C(22)—C(23)—H(23)	118.848
N(4)—C(20)—C(19)	118.97 (11)	C(24)—C(25)—H(25)	120.175
N(4)—C(20)—C(21)	119.55 (11)	C(26)—C(25)—H(25)	120.176
C(19)—C(20)—C(21)	121.48 (12)	C(25)—C(26)—H(26)	119.375
C(20)—C(21)—C(22)	119.81 (12)	C(27)—C(26)—H(26)	119.367
C(17)—C(22)—C(21)	120.63 (12)	C(26)—C(27)—H(27)	119.913
C(17)—C(22)—C(23)	120.58 (11)	C(28)—C(27)—H(27)	119.923
C(21)—C(22)—C(23)	118.78 (12)	C(28)—C(30)—H(30)	120.419
N(5)—C(23)—C(22)	122.27 (12)	C(31)—C(30)—H(30)	120.408
N(5)—C(24)—C(25)	124.01 (11)	C(30)—C(31)—H(31)	120.222
N(5)—C(24)—C(29)	115.21 (11)	C(32)—C(31)—H(31)	120.227
C(25)—C(24)—C(29)	120.76 (12)	N(6)—C(32)—H(32)	118.204
C(24)—C(25)—C(26)	119.65 (12)	C(31)—C(32)—H(32)	118.209
			
O(2)—N(1)—C(4)—C(3)	−2.84 (16)	C(11)—C(12)—C(13)—C(8)	0.56 (17)
O(3)—N(1)—C(4)—C(5)	−1.75 (16)	C(13)—C(12)—C(14)—C(15)	0.26 (17)
C(7)—N(2)—C(8)—C(9)	−0.65 (19)	C(14)—C(12)—C(13)—N(3)	0.26 (18)
C(13)—N(3)—C(16)—C(15)	0.39 (17)	C(12)—C(14)—C(15)—C(16)	−0.43 (18)
C(16)—N(3)—C(13)—C(12)	−0.57 (17)	C(14)—C(15)—C(16)—N(3)	0.10 (19)
O(4)—N(4)—C(20)—C(19)	0.75 (17)	O(6)—C(17)—C(22)—C(23)	1.31 (20)
O(5)—N(4)—C(20)—C(21)	1.07 (18)	C(18)—C(17)—C(22)—C(21)	1.45 (18)
C(23)—N(5)—C(24)—C(25)	1.0 (2)	C(22)—C(17)—C(18)—C(19)	−2.17 (19)
C(29)—N(6)—C(32)—C(31)	−0.54 (19)	C(17)—C(18)—C(19)—C(20)	1.1 (2)
C(32)—N(6)—C(29)—C(28)	0.14 (18)	C(18)—C(19)—C(20)—C(21)	0.84 (20)
O(1)—C(1)—C(6)—C(7)	−2.14 (18)	C(19)—C(20)—C(21)—C(22)	−1.53 (20)
C(2)—C(1)—C(6)—C(5)	−3.13 (17)	C(20)—C(21)—C(22)—C(17)	0.32 (19)
C(6)—C(1)—C(2)—C(3)	2.60 (17)	C(17)—C(22)—C(23)—N(5)	−0.1 (2)
C(1)—C(2)—C(3)—C(4)	−0.08 (18)	N(5)—C(24)—C(29)—N(6)	−0.88 (17)
C(2)—C(3)—C(4)—C(5)	−2.11 (18)	C(25)—C(24)—C(29)—C(28)	0.72 (18)
C(3)—C(4)—C(5)—C(6)	1.54 (18)	C(29)—C(24)—C(25)—C(26)	−0.71 (19)
C(4)—C(5)—C(6)—C(1)	1.17 (18)	C(24)—C(25)—C(26)—C(27)	0.00 (20)
C(1)—C(6)—C(7)—N(2)	0.57 (19)	C(25)—C(26)—C(27)—C(28)	0.69 (20)
N(2)—C(8)—C(13)—N(3)	0.29 (16)	C(26)—C(27)—C(28)—C(29)	−0.67 (19)
C(9)—C(8)—C(13)—C(12)	−0.22 (17)	C(27)—C(28)—C(29)—C(24)	−0.02 (18)
C(13)—C(8)—C(9)—C(10)	−0.30 (18)	C(29)—C(28)—C(30)—C(31)	−0.49 (18)
C(8)—C(9)—C(10)—C(11)	0.49 (18)	C(30)—C(28)—C(29)—N(6)	0.37 (19)
C(9)—C(10)—C(11)—C(12)	−0.14 (18)	C(28)—C(30)—C(31)—C(32)	0.15 (20)
C(10)—C(11)—C(12)—C(13)	−0.39 (18)	C(30)—C(31)—C(32)—N(6)	0.4 (2)

Symmetry codes: (i) *x*−1, *y*, *z*; (ii) −*x*+1, −*y*+1, −*z*; (iii) −*x*, −*y*+1, −*z*; (iv) *x*+1, *y*, *z*; (v) −*x*+1, −*y*+2, −*z*; (vi) −*x*+2, −*y*+1, −*z*; (vii) −*x*+2, −*y*+2, −*z*; (viii) −*x*, −*y*+1, −*z*+1; (ix) −*x*, −*y*+2, −*z*+1; (x) −*x*+1, −*y*+1, −*z*+1; (xi) −*x*+1, −*y*, −*z*+1; (xii) *x*−1, *y*, *z*+1; (xiii) *x*+1, *y*, *z*−1; (xiv) *x*, *y*+1, *z*; (xv) *x*, *y*−1, *z*; (xvi) *x*−1, *y*+1, *z*; (xvii) *x*+1, *y*−1, *z*.

### Hydrogen-bond geometry (Å, °)

**Table:**

*D*—H···*A*	*D*—H	H···*A*	*D*···*A*	*D*—H···*A*
N2—H2N···O1	0.880	2.013	2.6849 (15)	132.26
N2—H2N···N3	0.880	2.305	2.6942 (16)	106.77
N5—H5N···O6	0.880	1.875	2.5915 (14)	137.26
N5—H5N···N6	0.880	2.246	2.6645 (15)	108.87
